# DuXplore: A Dual-Hierarchical Deep Learning Model for Prognostic Prediction of Hepatocellular Carcinoma in Digital Pathology

**DOI:** 10.3390/diagnostics15232981

**Published:** 2025-11-24

**Authors:** Haotian Zhang, Mengling Liu, Xinshen Zhao, Yichen Zhang, Li Sui

**Affiliations:** 1 School of Health Science and Engineering, University of Shanghai for Science and Technology, Shanghai 200093, China17521018375@163.com (X.Z.); 2 Department of Biology, University of Pennsylvania, Philadelphia, PA 19104, USA

**Keywords:** hepatocellular carcinoma, whole slide image, survival risk prediction, tissue interaction modeling

## Abstract

**Background**: Spatial heterogeneity in tumor tissue has been linked to patient prognosis. To exploit both structural and semantic cues in whole slide images (WSIs), we propose Dual eXplanatory Framework (DuXplore), a dual-branch deep learning framework that integrates tissue architecture and cellular morphology for hepatocellular carcinoma (HCC) prognosis. **Method**: At the macroscopic level, DuXplore constructs a multi-channel tissue organization probability map (MTOP) to represent the spatial layout of eight tissue categories within the WSIs. At the microscopic level, a feature-guided Fused Structure Tensor (FST) based on tissue composition is employed to extract representative cell morphology patches. Accordingly, MTOP representations are modeled by Macro-Net, while FST-guided patches are modeled by Micro-Net. Each branch produces a 32-dimensional prognostic embedding, which are fused and passed through a multi-layer perceptron with a Cox proportional hazards head to generate patient-level risk predictions. To further elucidate the distinct contributions of the two branches, we conducted model-agnostic interpretability analyses, including occlusion sensitivity mapping (OSM) on MTOP and nuclear morphometrics from CellProfiler on high- versus low-risk tiles. **Result**: DuXplore achieves promising performance with C-indices of 0.764 on the public Cancer Genome Atlas (TCGA) dataset and 0.713 on the Eastern Hepatobiliary HCC (EHBH) cohort from our clinical center, along with significant patient risk stratification (log-rank *p* < 0.001). OSM highlighted necrosis and central fibrosis as high-risk and marginal fibrosis as protective; these patterns were corroborated by multivariable Cox using reproducible structural parameters (N-ratio, FIB-center, FIB-edge). Micro-level analysis revealed that higher nuclear staining intensity, increased texture irregularity (GLCM features), and greater morphological heterogeneity characterize high-risk tiles, aligning with pathological understanding. **Conclusions**: DuXplore advances prognostic modeling by coupling structure-aware micro-sampling with macro architectural encoding, delivering robust, generalizable survival prediction and biologically plausible explanations. While validated on HCC WSIs, broader multi-center, multi-omics studies are warranted to refine sampling scales and enhance clinical translation.

## 1. Introduction

In recent years, studies have revealed that the spatial interaction patterns among different tissue components within tumor regions not only reflect underlying biological heterogeneity but also have significant prognostic implications. For instance, patients with head and neck squamous cell carcinoma exhibiting close spatial proximity between PD-1^+^ T cells and PD-L1^+^ tumor cells respond better to immunotherapy [1]. In metastatic melanoma and osteosarcoma, the co-occurrence frequency of immune cells with antigen-presenting or immunosuppressive cells has also been shown to correlate significantly with survival outcomes [2,3]. Moreover, in non-small cell lung cancer and combined hepatocellular–cholangiocarcinoma, the proportional areas of different tissue types can effectively distinguish patient prognosis subgroups [4,5]. These findings collectively suggest that intra-tumoral tissue interactions are a critical source of prognostic information.

As the gold standard in digital pathology, WSIs [6] offer unique advantages in preserving structural integrity and spatial continuity, thereby capturing authentic spatial distributions and interaction patterns of tumor tissues. However, the enormous size of WSIs presents computational challenges for downstream modeling. Thus, a key issue in high-throughput pathology analysis is how to compress WSIs effectively while retaining their essential structural and interaction information.

Weakly supervised survival prediction from WSIs is currently routinely formulated as a multiple-instance learning (MIL) problem. The CLAM model [7], originally proposed for subtype classification, was re-trained with its attention-based pooling module paired with a Cox head, achieving a competitive C-index on more than 3000 TCGA slides. Similarly, the TransMIL model [8] has been adapted for survival modeling tasks, with its transformer self-attention architecture validated on 11,000 TCGA cases to establish the state-of-the-art (SOTA) performance in the pan-cancer field. Additionally, the DeepSurv model [9], which utilizes a Cox proportional hazards deep neural network, has also contributed significantly to personalized survival predictions. Although both models can generate slide-level representations, they still treat image patches as independent instances and overlook the inherent hierarchical organization of tissues—this limitation serves as the core motivation for our integration of explicit structural priors into deep survival modeling.

While most existing survival models overlook the spatial organization of tissues, a few recent studies have explicitly incorporated structural priors into WSI-based prognostic modeling. One representative example is PathFinder [10], which models WSIs at a macroscopic level by constructing multi-class tissue distribution maps and applying attribution analysis to link specific tissue structures—such as necrosis and invasive margins—to adverse outcomes. Although this approach successfully captures architectural-level prognostic cues, it neglects fine-grained cellular patterns that are often critical in digital pathology. In contrast, another line of research has introduced dual-branch frameworks that jointly model macroscopic architectural features and microscopic semantic features [11]. In these designs, a macroscopic branch encodes tissue-level organization, while a microscopic branch extracts cellular representations from tiled patches, enabling multi-scale feature fusion. Although these frameworks have improved over earlier purely patch-based models, their microscopic sampling strategies remain largely empirical, typically based on simple rules such as random or uniform selection rather than structure-informed design, meaning that the micro-level representation is not fully informed by the underlying structural priors.

To address these limitations, we propose a structure-aware patch sampling strategy for the microscopic branch. Building on the macroscopic pipeline, this strategy performs stratified patch extraction based on eight predefined tissue categories, enhancing the microscopic branch’s ability to capture structural context and generalize across WSIs. Based on this approach, we further develop DuXplore—a dual-branch deep learning model that combines macroscopic structural features with microscopic cellular representations.

Evaluated on both the public TCGA dataset [12] and a private clinical cohort from our institution, DuXplore demonstrates robust prognostic performance, achieving C-index scores of 0.764 and 0.712, respectively. Notably, without incorporating any additional clinical variables, the model effectively stratifies patients into distinct risk groups. Kaplan–Meier survival analysis [13] reveals statistically significant differences between the high- and low-risk groups (log-rank *p* < 0.01).

## 2. Materials and Methods

### 2.1. Data Acquisition

We evaluated the proposed method on two independent cohorts of patients diagnosed with HCC. The first cohort was derived from TCGA project. WSIs and associated clinical annotations were obtained via TCGA Data Portal, while survival information, including overall survival (OS) time, was supplemented through the UCSC Xena platform. The second cohort, referred to as the EHBH dataset, is a proprietary single-center database constructed at our institution. All patients were pathologically confirmed with primary HCC and underwent curative surgical resection, with complete clinical profiles and follow-up survival data. The WSIs were digitized using the KF-PRO-120-HI scanner (Jiangfeng Bio, Jinhua, China), and the follow-up duration ranged from 0.167 to 290 months, providing comprehensive long-term survival information.

During image preparation, we excluded slides exhibiting staining artifacts, blurring, tissue folding, missing survival data, or lacking coverage of the tumor core. For patients with multiple WSIs, an experienced pathologist selected the most representative slide encompassing the tumor core for subsequent analysis. All selected slides were stained with hematoxylin and eosin (H&E) [14].

After quality control, the final dataset included 172 WSIs from 172 patients in TCGA cohort and 167 WSIs from 167 patients in the EHBH cohort.

### 2.2. Macro–Micro Encoding

**Figure 1:**
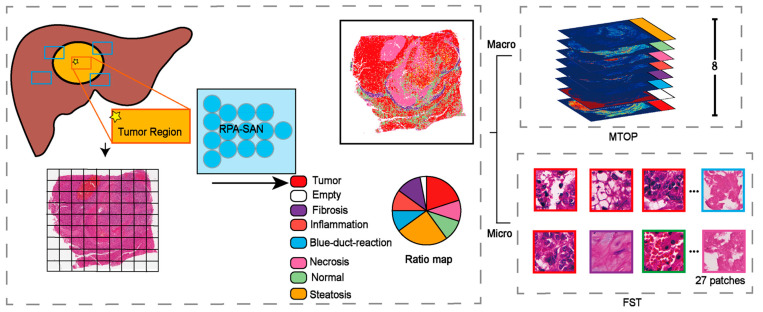
Macro-Micro Input Encoding. MTOP, multi-channel tissue organization probability map; FST, Fused Structure Tensor.

Although WSIs provide ultra-high-resolution information at both tissue and cellular scales, their massive data volume poses significant computational challenges. Direct modeling at the cellular scale is not only computationally expensive but also prone to redundancy and feature noise. To address this, we propose a tissue-structure-guided dual-scale abstraction strategy that captures pathological features from both macroscopic and microscopic spatial perspectives, as illustrated in Figure 1.

#### 2.2.1. MTOP

Transfer learning [15] has demonstrated remarkable effectiveness in deep learning tasks in recent years. Prior studies have shown that models trained on large-scale tissue classification tasks can generalize well to similar domains without requiring further fine-tuning. Leveraging this advantage, we adopted the PathFinder network, pre-trained on a large-scale tissue classification dataset, to perform rapid and accurate tissue structure recognition. This approach helps mitigate the risk of classification errors due to limited sample sizes.

Specifically, WSIs from both TCGA and the EHBH cohorts were first preprocessed via background removal using the OTSU thresholding [16] and color normalization via the Vahadane method [17]. The processed WSIs were then divided into fixed-size image patches $I_{i,j}$ at a magnification of 20×. Each patch was fed into the PathFinder model to predict its tissue category, encoded as:

(1) $$z_{\left(i,j\right)}=argmax(PathFinder\left(I_{i,j}\right)),\;\;\;\;\;\;\;z\in R^{8}$$

Based on the predicted tissue labels, we constructed spatial channel maps $M_{z}$ for each tissue category within a downsampled spatial grid. This allowed us to compress the high-resolution distribution of tissue labels into a compact 2D spatial representation, defined as:

(2) $$M_{z}\left(p,q\right)=\left\{\begin{array}{cc} 1,\;\;\;\;\;\; & \mathrm{if}\;z_{\left(i,j\right)}=z\;and\;\left(p,q\right)=\left(\frac{i}{L},\frac{j}{W}\right),\;\;\;\;\;\;{\;M}_{z}\in R^{\frac{m}{L}\times \frac{n}{W}}\\ 0,\;\;\;\;\;\; & \mathrm{otherwise} \end{array}\right.$$

where $\left(p,q\right)$ denotes the coordinates after downsampling, m,n are the dimensions of the original WSI, and L,W are the horizontal and vertical downsampling factors, respectively.

By concatenating all eight tissue-specific channel maps, we generated the final MTOP:

(3) $$M=Concat\left(M_{1},M_{2},\ldots ,M_{8}\right),\;\;\;\;\;\;\;\;M\in R^{\frac{m}{L}\times \frac{n}{W}\times 8}$$

#### 2.2.2. FST

We designed a Fused Structure Tensor based on the spatial composition of tissue types within each WSI. Specifically, we first computed the proportion $r_{z}$ of each tissue category $z\in \left[0.7\right]$ across the entire WSI, defined as:

(4) $$r_{z}=\frac{\sum_{i,j}\;1(z_{\left(i,j\right)}=z)}{\sum_{z^{\prime }=0}^{7}\;\sum_{i,j}\;1(z_{\left(i,j\right)}=z^{\prime })},\;\;\;\;\;\;\;for\;z\;\in [0,7]$$

where $r_{z}$ denotes the spatial proportion of the *z*-th tissue type in the whole WSI.

Based on the computed proportions $r_{z}$, we performed category-wise stratified sampling over all patches in the WSI. For each patient, a total of N=27 patches were sampled, with the number of patches drawn from each tissue category z determined as:

(5) $$n_{z}=\left\lfloor r_{z}\cdot N\right\rfloor \;and\sum_{z=0}^{7}\;n_{z}=27$$

Here, $n_{z}$ represents the number of patches sampled from the *z*-th tissue type. If some tissue types occupy extremely small proportions and do not provide enough patches, the shortfall is compensated by sampling additional patches from dominant tissue types.

The final set of sampled patches is denoted as:

(6) $$\left\{I^{\left(1\right)},I^{\left(2\right)},\ldots ,I^{\left(27\right)}\right\},\;\;\;\;\;\;\;\;\;I^{\left(k\right)}\in R^{3\times L\times W}$$

All selected patches are concatenated along the channel dimension in their sampling order to construct a unified input representation for the microscopic branch (FST):

(7) $$FST=Concat\left(I^{\left(1\right)},I^{\left(2\right)},\ldots ,I^{\left(27\right)}\right),\;\;\;\;\;\;\;FST\in R^{3\times 27\times L\times W}$$

### 2.3. Establishment of DuXplore

**Figure 2:**
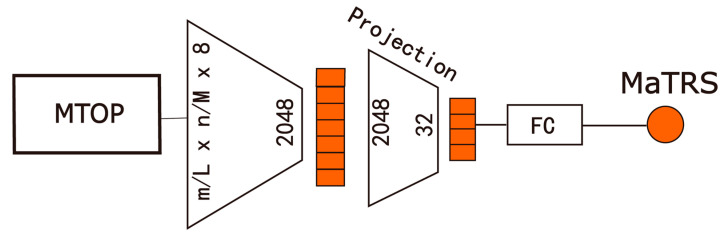
Macro-Net architecture. FC, Fully Connection layer; MaTRS, macro tumor risk score.

We independently constructed two prognostic models based on macroscopic structural features and microscopic semantic features, respectively, and conducted a systematic performance comparison. For the macroscopic branch model (Macro-Net), as illustrated in Figure 2, the input is the MTOP for each patient, with the prediction targets being the overall survival time and censoring status.

The model is built upon a ResNet50 backbone [18], with the input channel dimension adapted to 8 to accommodate the eight tissue types. The overall architecture consists of three main components: an Encoder, a Feature Projection Module, and a Risk Prediction Head.

Specifically, the input image $M\in R^{\frac{m}{L}\times \frac{n}{W}\times 8}$ is first processed by the encoder to extract spatial features:

(8) $$K_{Macro\_2048}=f_{Macro\_encoder}\left(M\right)$$

where $f_{Macro\_encoder}$ denotes the encoder network (ResNet50), and $K_{Macro\_2048}$ represents the resulting 2048-dimensional feature vector.

This high-dimensional representation is then compressed into a 32-dimensional vector via a fully connected layer:

(9) $$h_{Macro\_32}=f_{projection}\left(K_{Macro\_2048}\right)=ReLU\left(BN\left(FC\left(K_{Macro\_2048}\right)\right)\right)$$

where $f_{projection}$ denotes the feature projection module, *FC* is the fully connected layer, *BN* is batch normalization, and *ReLU* is the activation function.

Finally, a linear prediction head generates the macroscopic structure-based risk score, referred to as Macro tumor risk score (MaTRS):

(10) $$MaTRS={head}_{Macro\_pred}\left(h_{Macro_{32}}\right)$$

**Figure 3:**
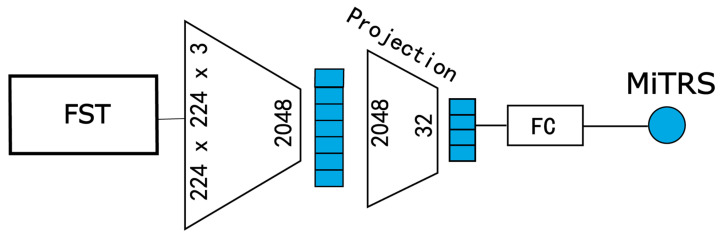
Micro-Net architecture. FC, Fully Connection layer; MiTRS, micro-level tumor risk score.

For the microscopic semantic branch model (Micro-Net), illustrated in Figure 3, we similarly operate at the patient level, but the input is replaced with a fine-grained patch set obtained via the FST. The goal is to generate a micro-level tumor risk score (MiTRS) for each patient.

This model is also built upon a modified ResNeXt50 backbone. The input channel number is adjusted to 81 (3 × 27) to accommodate the concatenated 27 RGB patches per patient. The overall architecture comprises three key components: an Encoder, a Feature Compression Module, and a Risk Prediction Head.

Formally, given the input image tensor $FST\in R^{3\times 27\times L\times W}$, the encoder first extracts spatial features:

(11) $$K_{Micro\_2048}=f_{Micro\_encoder}\left(FST\right)$$

where $f_{Micro\_encoder}$ denotes the encoder network, and $K_{Micro\_2048}$ is the resulting 2048-dimensional feature representation.

Next, the high-dimensional feature is projected into a compact 32-dimensional embedding via a fully connected compression layer:

(12) $$h_{Micro\_32}=f_{Micro\_comp}\left(K_{Micro\_2048}\right)=ReLU\left(BN\left(FC\left(K_{Micro\_2048}\right)\right)\right)$$

where is $f_{projection}$ the feature compression module; *FC*, *BN*, and *ReLU* represent the fully connected layer, batch normalization, and activation function, respectively.

Finally, a linear prediction head generates the MiTRS.

To further integrate the complementary strengths of macroscopic structural and microscopic semantic information for enhanced survival risk prediction, we developed a unified prognostic framework—DuXplore.

Specifically, within the DuXplore architecture, we removed the original prediction heads from both Macro-Net and Micro-Net, retaining only their respective feature extraction backbones. The extracted macroscopic and microscopic features are then jointly modeled and fed into a unified fusion prediction head, which outputs a comprehensive patient-level tumor risk score (PaTRS).

#### Optimization Objective

To perform survival risk prediction, we adopted the Cox proportional hazards model as the output layer, enabling semi-parametric modeling of censored survival data. During training, we optimized the model using the negative log partial likelihood [19], formulated as:

(13) $$L_{loss}=-\sum_{i:\delta_{i}=1}\left(\hat{h}-log\sum_{j\in R\left(t_{i}\right)}e^{\hat{h_{j}}}\right)$$

where $\hat{h}$ denotes the predicted PaTRS for the *i*-th patient, $\delta_{i}$ is the censoring indicator, $t_{i}$ is the observed survival time, and $R\left(t_{i}\right)$ represents the risk set, the set of patients still at risk at time $t_{i}$.

### 2.4. Performance Comparative Evaluation

To ensure a fair and reproducible comparison across different survival prediction frameworks, all models were trained and evaluated under a unified data processing and training protocol. The same patient-level data split was applied to all methods, with five-fold cross-validation conducted on the internal cohort to assess model stability and an independent external cohort used for generalization testing. For all models, WSIs were divided into non-overlapping patches of 224 × 224 pixels at 20× magnification, and identical preprocessing procedures were applied to ensure consistency across datasets.

We compared four representative paradigms of WSI-based survival modeling, including DeepSurv, CLAM, TransMIL, and PathFinder. In DeepSurv, a ResNet-50 network was used as the feature extractor before Cox proportional hazards modeling. All models were trained from scratch for up to 100 epochs using the Adam optimizer [20] with an initial learning rate of 1 × 10^−4^. A learning rate decay strategy was applied, reducing the learning rate by a factor of 0.1 when the validation performance plateaued. L2 regularization with a weight decay coefficient of 1 × 10^−5^ was used to mitigate overfitting [21]. To ensure robust evaluation, we conducted all experiments using 5-fold stratified cross-validation, which was also used for hyperparameter tuning.

### 2.5. Explanation of the Decision-Making Process of the Model

To explore the discriminative basis of Macro-Net, we first employed OSM [22] to locally occlude MTOP and generate risk-sensitive heatmaps, where red/blue represent higher/lower mortality risk, respectively.

Pathologists observed three distinct patterns in these visualizations: necrotic areas consistently signaled high risk; fibrosis within tumors indicated risk propensity; while fibrosis at tumor margins exhibited protective effects. Based on this observation, we hypothesize that necrosis and central fibrosis may correlate with poor prognosis, whereas marginal fibrosis may confer protective benefits.

To test this hypothesis, we formalized these regional features into three structural parameters: Necrotic area ratio (N-ratio), Tumor margin fibrosis area (FIB-center), and Tumor interior fibrosis area (FIB-edge). Area proportions were obtained via pixel counting, with edge regions defined by distance transformation and maintained at a fixed thickness. These parameters were subsequently incorporated into Cox regression analyses to quantify their relationship with survival outcomes.

To interpret the patch-level features received by Micro branch that correlate with prognosis, we applied CellProfiler 2.1.0 to automatically extract quantified image vectors from high- and low-risk tiles. We used CellProfiler 2.1.0 to identify and segment tumor cell nuclei within each tile, then measured the shape, intensity, and texture of the nuclear area for each nucleus. A total of 732 feature vectors were extracted, excluding null values and normalized to describe the aggregated morphology of tumor nuclei within the tile. Each feature vector contained nuclear information including mean, median, and standard deviation of nuclear size; contour length; orientation; ellipticity; texture entropy; centroid moment; and other metrics.

### 2.6. Statistical Analysis

All statistical analyses were performed using R software (version 4.2.2) and Python (version 3.8). Model performance was evaluated from three complementary perspectives. Discrimination was measured by Harrell’s concordance index (C-index) [23], quantifying each model’s ability to correctly rank patient survival times. Calibration was assessed using the Integrated Brier Score (IBS) [24], evaluating agreement between predicted and observed survival probabilities over time. Prediction accuracy was quantified by the inverse probability of censoring–weighted mean absolute error (IPCW-MAE), reflecting the absolute difference between predicted and observed survival times while accounting for censoring.

All metrics were computed under the same five-fold cross-validation protocol. For each model and metric, results are reported as the mean and 95% confidence interval (95% CI) estimated across folds using a t-based interval (df = 4). For the C-index, pairwise comparisons between two models were conducted using paired *t*-tests on fold-wise scores, and overall differences among multiple models were assessed using one-way repeated-measures ANOVA across folds. The same fold-wise procedure was applied to the IBS and IPCW-MAE. On the independent external cohort, we report point estimates obtained from the model selected by validation performance to characterize generalization.

Kaplan–Meier survival analysis was used to estimate survival probabilities, and log-rank tests were employed to assess statistical differences between risk groups [25]. Multivariate Cox regression analysis was conducted to identify independent variables significantly associated with overall survival.

All statistical tests were two-sided, and a *p*-value < 0.05 was considered statistically significant.

## 3. Results

### 3.1. Comparative Study of Input Stragety in Macro-Micro Encoding

To systematically evaluate the impact of different input configurations on survival prediction performance and to identify the optimal settings for DuXplore, we conducted a series of comparative input strategy experiments in both the Macro-Branch and Micro-Branch components.

**Figure 4:**
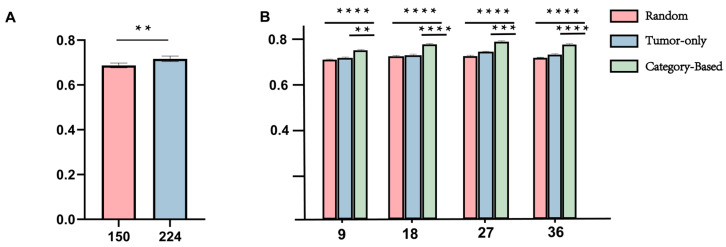
Encoding-Strategy Sampling. (**A**) Comparison of Macro Branch Strategies under five-fold cross-validation; (**B**) Macro Branch Strategies under five-fold cross-validation. **: *p* < 0.01, ***: *p* < 0.001, ****: *p* < 0.0001.

In the Macro-Encoding module, we compared two patch sizes—150 × 150 and 224 × 224—for constructing multi-channel tissue structure maps. Under identical network architectures and training setups, all images were converted into 8-channel structural maps based on dominant tissue categories and fed into the ResNet backbone for Cox-based survival modeling. As shown in Figure 4A, the 224 × 224 configuration achieved a significantly higher average C-index of 0.717 [95% CI: 0.692–0.741], compared to 0.686 [95% CI: 0.649–0.724] for the 150 × 150 setting (*p* < 0.05). These results suggest that larger patch sizes help better capture structural topology and improve the model’s discriminative ability in survival risk prediction.

For the Micro-Encoding module, we systematically evaluated the effect of patch sampling strategy and patch quantity on model performance. Using the TCGA cohort, we designed three sampling strategies: Global Random Sampling (Random), Tumor-Only Sampling, and Category-Based Sampling. Each was tested under four sampling sizes: 8, 16, 27, and 36 patches per patient. As illustrated in Figure 4B, the Category-Based strategy with 27 patches yielded the best performance, achieving an average C-index of 0.710 [95% CI: 0.682–0.737], consistently outperforming other settings. In contrast, Random and Tumor-Only strategies exhibited higher performance variability and significantly lower C-indices (*p* < 0.001).

Based on these results, we selected the Macro-Branch configuration using 224 × 224 structural patches and the Micro-Branch configuration using 27 category-guided patches as the optimal input settings for the final DuXplore framework.

### 3.2. Performance of DuXplore

DuXplore was implemented using its optimal input configuration, consisting of the MTOP constructed with 224 × 224-sized patches and the FST composed of 27 category-guided patches. By integrating multi-scale information within a unified feature space, DuXplore generates patient-level prognostic risk scores for tumor outcome assessment.

**Figure 5:**
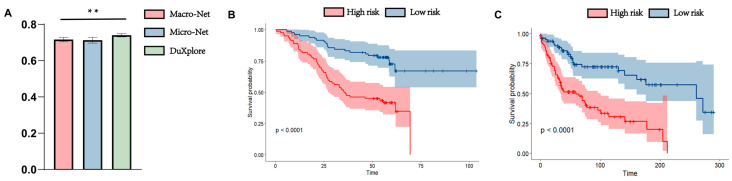
Performance of the DuXplore (**A**) Comparison of the three model architectures under five-fold cross-validation; (**B**) Kaplan–Meier survival analysis of high- and low-risk groups in TCGA cohort, stratified by the median PaTRS score; (**C**) Kaplan–Meier survival analysis on the external EHBH cohort without modifying the parameters. Shaded areas in panels (**B**,**C**) represent the confidence intervals of the survival curves. **: *p* < 0.01.

We compared the prognostic performance of three model architectures under their respective optimal input settings. As shown in Figure 5A, DuXplore consistently achieved the highest C-index across all cross-validation folds, with an average of 0.764 [95% CI: 0.746–0.782], significantly outperforming both the Macro branch (average C-index = 0.717) and Micro branch (average C-index = 0.710) (*p* < 0.01). These experiments were conducted on the TCGA cohort.

To evaluate generalizability, we directly transferred the trained DuXplore model to the independent EHBH cohort without any fine-tuning. The model achieved a C-index of 0.713 on EHBH, confirming its robust performance.

We further conducted Kaplan–Meier survival analysis based on the DuXplore-generated PaTRS scores. Patients in each cohort were stratified into high-risk and low-risk groups according to the median PaTRS score. In TCGA cohort, patients in the high-risk group had significantly worse survival outcomes compared to the low-risk group (*p* < 0.001, Figure 5B). A consistent trend was observed in the independent EHBH cohort, where the high-risk group also exhibited significantly poorer survival (*p* < 0.001, Figure 5C).

To assess the clinical relevance of PaTRS, we performed univariate and multivariate Cox regression analyses on the TCGA cohort. In univariate analysis, both PaTRS (*p* < 0.001, HR = 3.87, 95% CI: 2.513–5.959) and T stage (T3/T4) were significantly associated with worse survival. In multivariate Cox analysis including clinical covariates such as TNM stage, gender, and race, only PaTRS remained statistically significant (*p* < 0.001, HR = 3.877, 95% CI: 2.386–6.302), suggesting it serves as an independent prognostic factor.

Similarly, in the EHBH external validation cohort, PaTRS maintained its statistical significance in both univariate (*p* < 0.001, HR = 3.097, 95% CI: 1.687–5.686) and multivariate analysis (*p* < 0.001, HR = 3.284, 95% CI: 1.735–6.216). Other clinical factors, such as age, gender, hepatitis status, antiviral treatment, smoking, alcohol consumption, and diabetes history, showed no statistical significance. Detailed results are presented in Tables S1–S4.

### 3.3. Comparative Evaluation with Other Survival Models

We further compared DuXplore with four representative deep learning survival models, DeepSurv, CLAM, TransMIL, and PathFinder, to evaluate relative performance across different modeling paradigms. As shown in Table 1, DuXplore achieved the highest discrimination on the TCGA cohort, with a mean C-index of 0.764 [95% CI: 0.746–0.782], exceeding all baseline approaches. PathFinder and TransMIL followed closely, reaching 0.752 [0.734–0.770] and 0.739 [0.721–0.757], respectively. In terms of calibration and absolute error, TransMIL obtained the lowest Integrated Brier Score (0.149 [0.143–0.155]), while PathFinder recorded the smallest IPCW-MAE (0.220 [0.210–0.230]). DuXplore yielded comparable scores (IBS = 0.152 [0.146–0.158], IPCW-MAE = 0.223 [0.213–0.233]), indicating balanced performance across all evaluation metrics.

When tested on the independent EHBH cohort (Table 2), DuXplore maintained the strongest overall discrimination with a C-index of 0.718, outperforming PathFinder (0.709) and TransMIL (0.702). It also achieved the lowest IPCW-MAE (0.226), reflecting accurate time-to-event estimation on unseen data. The Integrated Brier Score remained competitive (0.159), slightly above that of TransMIL (0.156) and PathFinder (0.157).

### 3.4. Explanation in Macro and Micro Branch

**Figure 6:**
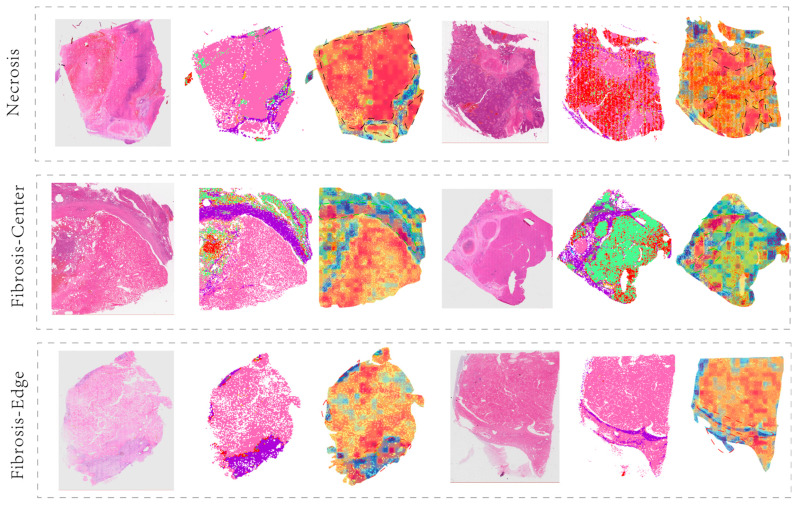
Explanation of Macro Branch. Each whole slide image (WSI) is presented in three formats: the original WSI (**left**), the global segmentation map (**middle**), and the merged image of OSM and the global segmentation map (**right**). The first row of images displays risk zones based on necrotic area distribution (indicated by black banded curves). The second row highlights the prognostic significance of intra-tumoral fibrosis (green banded curves). The third row demonstrates the prognostic value of tumor margin fibrosis, contrasting with intra-tumoral fibrosis (red banded curves). High-risk (masked in red) and low-risk (masked in blue).

OSM visualization revealed distinct responses of Macro-Net across different tissue regions: Necrotic areas consistently exhibited strong high-risk signals, while fibrosis at tumor margins primarily contributed low risk, and fibrosis within the tumor core emerged as high-risk zones (see Figure 6). This phenomenon suggests that central and peripheral fibrosis may exert opposing prognostic effects.

Building on this, we quantified and incorporated only three predefined structural parameters into Cox multivariate regression within the EHBH dataset: N-ratio, FIB-edge, and FIB-center. The effect directions of the analysis results were consistent with visual observations (see Table 3): both N-ratio and FIB-center showed positive associations with poor outcomes, while FIB-edge demonstrated a protective association with better outcomes (HR < 1). In summary, Macro-Net not only highlights key regions associated with prognosis in OSM but also provides statistical validation through the aforementioned reproducible macrostructural indicators.

**Figure 7:**
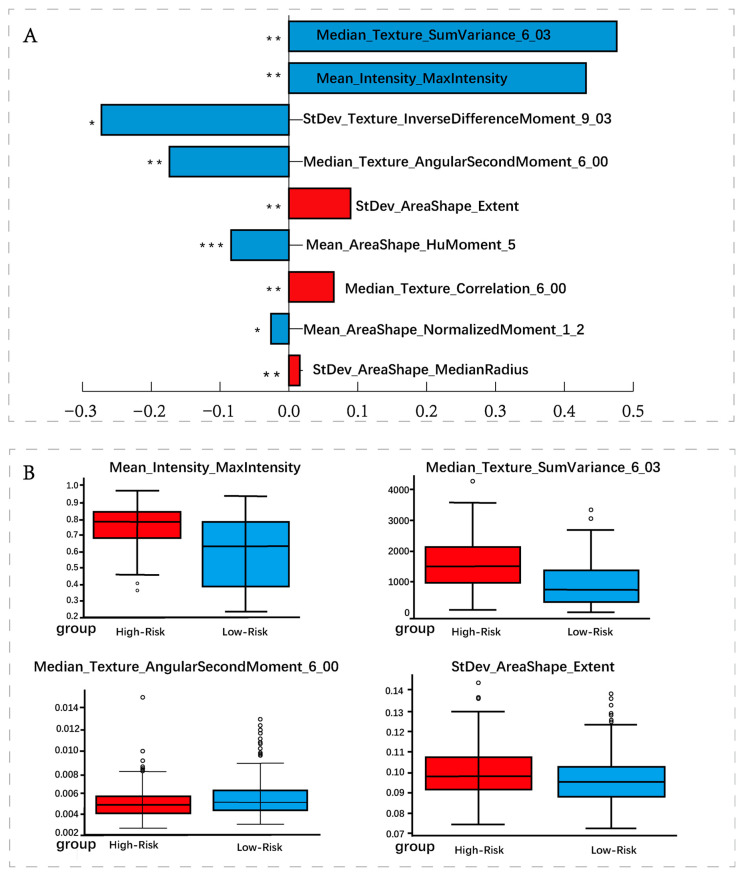
Explanation of Micro Branch (**A**) The names and coefficients of the most significant measurements that correlated with TiRS after lasso; (**B**) Inter-group differences and the intersection features of lasso-related vectors. * *p* < 0.05; ** *p* < 0.01; *** *p* < 0.001.

To validate the interpretability of risk assessments generated by the Micro branch, we focused our analysis on nuclear morphology. Utilizing CellProfiler, we extracted quantifiable nuclear-level features to reveal the discriminative basis of the Micro branch. Specifically, we sampled tissue sections from patients in the top 10% and bottom 10% of MiTRS scores and extracted 732 nuclear morphological features spanning dimensions such as size, shape, texture, and staining intensity. We then applied a dual-criterion feature selection process: First, Lasso regression was applied for feature dimensionality reduction, identifying candidate factors most correlated with risk scores (Figure 7A). Second, Mann–Whitney U tests compared distribution differences between high- and low-risk groups to identify significantly differential features. Finally, the intersection of these two steps yielded key features satisfying both “correlation with scores” and “significant intergroup differences” (Figure 7B).

Results indicate the most representative measurements include maximum staining intensity of cell nuclei, median variance of grayscale co-occurrence matrix (GLCM) for nuclear texture, median of GLCM second-order moments, and standard deviation of the ratio between nuclear area and bounding rectangle area. In other words, nuclei in high-risk sections exhibit higher staining intensity, more pronounced textural irregularities and granularity, and greater morphological variability; conversely, low-risk sections demonstrate textural orderliness and consistent nuclear morphology. Representative visualization panels illustrating these morphological differences between high- and low-risk regions have been provided in Supplementary Figure S1.

Collectively, these findings indicate that micro branch captures fine-grained features consistent with pathological knowledge.

## 4. Discussion

Although existing prognostic models have formally achieved dual-branch collaborative modeling of structural and fine-grained information, their fine-grained information selection strategies often fail to comprehensively reflect the complexity of the tumor microenvironment. This limitation may cause models to overlook critical information when generalizing to heterogeneous patient populations.

Methodologically, we propose a structure-aware sampling strategy for the micro-branch within the dual-branch collaborative network to comprehensively capture fine-grained features of the tumor microenvironment. To determine an appropriate sampling scale, we conducted a series of comparative experiments using 9, 18, 27, and 36 patches per case. As shown in Figure 5B, the Category-based strategy consistently outperformed both Random and Tumor-only sampling across all settings and achieved the best stability and overall performance under the 27-sample configuration (average C-index = 0.7097, *p* < 0.001). Notably, while previous studies typically adopt around 16 patches per case, our cross-sectional analysis empirically optimized the sampling quantity, revealing that excessive or insufficient patch numbers both reduce efficiency and stability. These findings indicate that the proposed structure-aware sampling not only enhances prognostic robustness but also maximizes the utilization efficiency of microenvironment information at an empirically optimized sampling scale.

To further evaluate the prognostic effectiveness of the proposed framework, we compared DuXplore with four representative deep survival models on both the TCGA and EHBH cohorts. Across all metrics, DuXplore demonstrated consistently strong discrimination, achieving the highest C-index on both datasets. On the TCGA cohort, DuXplore achieved a mean C-index of 0.764 [95% CI: 0.746–0.782], outperforming all baselines, while TransMIL and PathFinder obtained slightly lower Integrated Brier Score and IPCW-MAE values, respectively. These performance differences are expected, as the C-index primarily reflects relative risk ranking, whereas IBS and MAE emphasize calibration and absolute time estimation. Models with global attention (TransMIL) or explicit topological constraints (PathFinder) may thus yield better calibration, while DuXplore’s multi-scale, structure-aware representation enhances ranking discrimination.

On the external EHBH cohort, DuXplore maintained the highest C-index (0.718) and the lowest IPCW-MAE (0.226), suggesting robust generalization across data distributions. Overall, these results highlight that integrating structural and semantic priors enables DuXplore to achieve a favorable balance between discrimination, calibration, and prediction accuracy.

However, despite the encouraging results achieved in predictive performance by the aforementioned methods, relying solely on performance improvements is insufficient to support the application of these models in clinical settings. In other words, even if a model achieves a high C-index, it does not fully demonstrate that the features relied upon for prediction are intrinsically linked to prognosis [26,27]. Therefore, this study further conducted a systematic interpretability analysis of both macro and micro branches to reveal the key pathological features relied upon by the model during prediction and to assess whether these features possess biological plausibility.

Structural features reflecting spatial interactions within WSIs were identified as key correlates for the macro branch’s prognostic modeling. Among these, the distribution of fibrotic regions appeared to provide informative cues for survival prediction rather than deterministic effects. Previous studies have similarly reported that fibrotic regions located within or surrounding tumors exhibit distinct prognostic orientations: those within tumors tend to correlate with poorer outcomes [28], whereas peri-tumoral fibrosis is generally associated with a more favorable prognosis [29]. Interestingly, even without explicit prior knowledge, the network appeared to capture these associations between tissue organization and prognosis, suggesting that the learned representations may implicitly encode biologically relevant spatial patterns. In parallel, prognostic indicators such as necrotic areas remain well-recognized by pathologists, yet their reliable quantification is still challenging within routine workflows.

By comparing nuclear texture features between high- and low-risk groups in Micro Branch, we found the model’s decision criteria align closely with traditional pathological understanding. Specifically, high-risk sections exhibit stronger nuclear staining intensity, more complex and irregular texture undulations, and greater morphological heterogeneity, whereas low-risk sections display more ordered texture structures and relatively consistent nuclear morphology. These features have long been associated with tumor cell malignancy and invasiveness. Thus, Micro Branch’s focus on these indicators demonstrates that it does not merely output risk scores as a “black box,” but rather captures biologically meaningful subtle clues.

In summary, this study represents an innovative methodological advancement in prognostic modeling for primary liver cancer. By introducing a structure-aware sampling strategy within the micro-branch of our dual-branch synergistic network, we enable the model to comprehensively capture fine-grained features of the tumor microenvironment while demonstrating superior and stable performance across multiple sampling scales. Furthermore, the complementary modeling of macro and micro branches enhances the model’s ability to comprehensively characterize both macroscopic tumor structure and local cellular morphology, achieving performance surpassing either branch alone. Moreover, most features in our dual-branch model remain readily interpretable for pathologists with relevant expertise. While our model does not prioritize maximizing accuracy at all costs, it demonstrates remarkable robustness and generalization when applied to validation samples.

Nevertheless, this study has certain limitations. The current framework focuses solely on histopathological information without integrating molecular or clinical modalities, which may restrict its overall generalizability. Moving forward, DuXplore can be naturally extended into a multimodal learning paradigm that jointly models histology, molecular profiles, and clinical covariates. Built upon its dual-branch design, additional modules could be introduced to encode transcriptomic or genomic representations, while clinical indicators such as AFP level, TNM stage, and vascular invasion could be integrated through late fusion or attention-based mechanisms. This multimodal extension would enable complementary information from biological and clinical domains to be leveraged, thereby improving predictive precision, interpretability, and clinical utility.

Furthermore, although the proposed structure-aware sampling strategy demonstrated robust advantages in prognostic prediction, its optimal sampling scale and generalizability require further validation across diverse cancer types and more complex pathological contexts. Future efforts should therefore focus on constructing multi-center, multi-modal datasets to comprehensively evaluate the robustness and adaptability of this framework. Additionally, refining structure-guided sampling mechanisms may further promote the clinical translation of prognostic modeling within the precision medicine paradigm.

## 5. Conclusions

In this work, we present DuXplore, a dual-branch prognostic framework that explicitly integrates macro-scale tumor architecture with micro-scale tumor-microenvironment interactions through a structure-aware sampling strategy. By empirically optimizing the number of patches to 27 per case, the micro-branch captures fine-grained morphological cues that are biologically aligned with established pathological criteria, while the macro-branch encodes spatial tissue organization that implicitly reflects clinically relevant stromal patterns. Extensive experiments on TCGA and the external EHBH cohort show that DuXplore achieves state-of-the-art discrimination (C-index 0.764 and 0.718, respectively) and maintains robust calibration across data distributions. Interpretability analyses further reveal that the model’s decisions are driven by histopathological features—fibrotic distribution, nuclear pleomorphism, and textural heterogeneity—that corroborate expert knowledge, bridging the gap between predictive power and biological plausibility.

Despite these advances, DuXplore is currently limited to histopathology; incorporation of genomic, transcriptomic, and clinical covariates constitutes a logical next step. Future work will extend the dual-branch design to a multimodal paradigm, validate the structure-aware sampling strategy across multiple cancer types and institutions, and refine guidance mechanisms for patch selection to accelerate clinical deployment. Collectively, our study demonstrates that principled integration of structural priors and microenvironmental detail can yield prognostic models that are not only highly accurate and generalizable but also interpretable and ready for translation into precision-oncology workflows.

## Figures and Tables

**Table 1:** Performance comparison of survival models on the TCGA cohort.

Model	C-Index	IBS	IPCW-MAE
DeepSurv	0.684[0.666–0.702]	0.174[0.167–0.181]	0.252[0.238–0.266]
CLAM	0.719[0.701–0.737]	0.161[0.154–0.168]	0.239[0.227–0.251]
TransMIL	0.739[0.721–0.757]	**0.149[0.143–0.155]**	0.232[0.221–0.244]
PathFinder	0.752[0.734–0.770]	0.151[0.145–0.157]	**0.219[0.211–0.231]**
DuXplore (Proposed)	**0.764[0.746–0.782]**	0.152[0.146–0.158]	0.223[0.213–0.233]

Values in brackets indicate the 95% confidence intervals. Bold values denote the best performance for each metric.

**Table 2:** Performance comparison of survival models on the EHBH cohort.

Model	C-Index	IBS	IPCW-MAE
DeepSurv	0.662	0.182	0.260
CLAM	0.689	0.168	0.244
TransMIL	0.702	**0.156**	0.236
PathFinder	0.709	0.157	0.231
**DuXplore (Proposed)**	**0.718**	0.159	**0.226**

The external validation set adopts point estimation. Bold values denote the best performance for each metric.

**Table 3:** Multivariable Cox for Structure parameter.

Covariate	Coef	Se_Coef	HR	Cl_Lower	Cl_Upper	*p*_Value
N-Ratio	0.2430	0.1001	1.2751	1.0480	1.5514	0.0152
FIB-Center	1.4127	0.6028	4.1070	1.2600	13.3863	0.0191
FIB-Edge	−0.2477	0.1097	0.7806	0.6295	0.9679	0.0240

## Data Availability

The original data presented in the training dataset of this document can be freely accessed at https://www.cancer.gov/ccg/research/genome-sequencing/tcga (accessed on the 11 August 2025). The data presented in this external validation set cannot be obtained at any time, as it is part of an ongoing research project. If you need to access these data, please make a request to Shanghai Eastern Hepatobiliary Surgery Hospital.

## Supplementary Materials

The following supporting information can be downloaded at: https://www.mdpi.com/article/10.3390/diagnostics15232981/s1, Figure S1: Visualization of morphological differences between high- and low-risk histological regions; Table S1: TCGA Univariate Cox regression; Table S2: TCGA Multivariate Cox regression; Table S3: EHBH Univariate Cox regression; Table S4: EHBH Multivariate Cox regression.

## Abbreviations

The following abbreviations are used in this manuscript:

MTOP	Multi-channel tissue organization probability map
FST	Fused Structure Tensor
WSI	Whole Slide images
DuXplore	Dual explanatory Framework
HCC	Hepatocellular carcinoma
TCGA	The public cancer Genome Atlas
OS	Overall Survival
EHBH	Eastern Hepatocellular HCC Dataset
H&E	Hematoxylin and Eosin
MaTRS	Macro tumor risk scores
MiTRS	Micro tumor risk scores
PaTRS	Patient level tumor risk scores
GLCM	Grayscale co-occurrence matrix
MIL	multiple-instance learning
SOTA	State-of-the-art
