# Supplementary material for: DuXplore: A Dual-Hierarchical Deep Learning Model for Prognostic Prediction of Hepatocellular Carcinoma in Digital Pathology
Source: Diagnostics (Basel). 2025 Nov 24;15(23):2981. doi: 10.3390/diagnostics15232981 (PMC12691227; doi:10.3390/diagnostics15232981)
Supplement: Supplementary file 1 [file diagnostics-15-02981-s001.zip › diagnostics-3911468-supplementary.pdf]

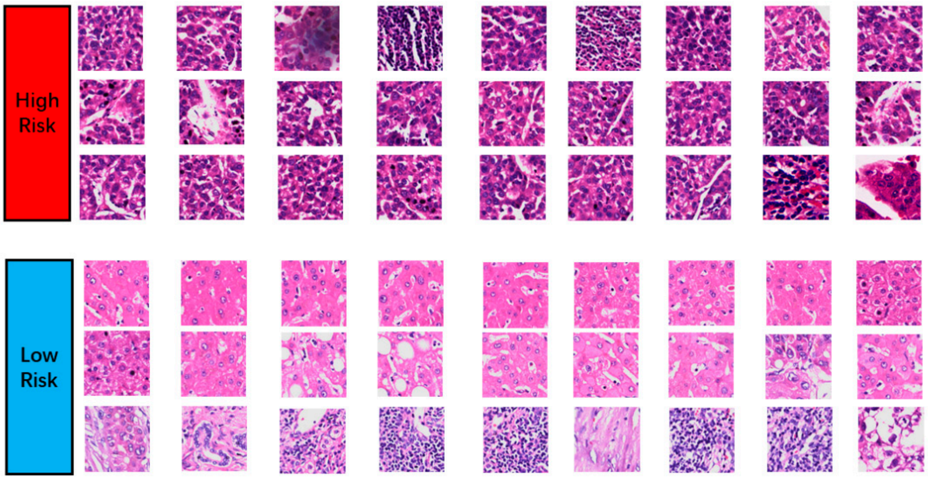

Figure S1 Visualization of morphological differences between high- and low-risk histological regions.

Table S1 TCGA Univariate Cox regression

Variable	HR	Cl_lower	Cl_upper	*p*
PaTRS	3.87	2.513	5.959	**8.00E-10**
T Stage				
T1	1			
T2	1.765	0.945	3.298	0.0746
T3	3.748	2.104	6.675	**7.26E-06**
T3a	2.509	0.947	6.652	0.0644
T3b	3.414	0.801	14.547	0.0969
T4	4.906	2.175	11.063	**0.000127**
N Stage				
N0	1			
N1	1.541	0.375	6.329	0.549
NX	1.539	0.841	2.814	0.162
M Stage				
M0	1			
M1	2.798	0.875	8.948	0.0828
MX	1.688	0.837	3.405	0.144
Race				
Asian	1			
White	0.81	0.5	1.311	0.391
black or african american	1.299	0.4	4.21	0.663
not reported	1.617	0.499	5.241	0.423
Gender				
Female	1			
Male	1.298	0.812	2.075	0.276

Abbreviations: HR, hazard ratio. Cl_lower, Lower Confidience Limit . Cl_upper, Upper Confidience Limit. PaTRS, Patient level tumor risk scores.

The p was calculated based on likelihood ratio test, it is significant at p <.005

Table S2 TCGA Multivariate Cox regression

Variable	HR	Cl_lower	Cl_upper	*p*
PaTRS	3.877	2.386	6.302	**4.55E-08**
T Stage				
T1	1			
T2	1.896	0.97	3.705	0.0614
T3	3.155	1.636	6.085	**0.000608**
T3a	2.648	0.969	7.24	0.0577
T3b	2.701	0.532	13.724	0.231
T4	2.473	0.838	7.299	0.101
N Stage				
N0	1			
N1	1.553	0.36	6.694	0.555
NX	1.784	0.668	4.767	0.248
M Stage				
M0	1			
M1	1.933	0.453	8.258	0.374
MX	1.193	0.388	3.668	0.758
Race				
Asian	1			
White	0.315	0.162	0.614	**0.000692**
black or african american	0.78	0.15	4.055	0.768
not reported	1.435	0.363	5.672	0.607
Gender				
Female	1			
Male	0.844	0.475	1.499	0.563

Abbreviations: HR, hazard ratio. Cl_lower, Lower Confidience Limit . Cl_upper, Upper Confidience Limit. PaTRS, Patient level tumor risk scores.

The *p* was calculated based on likelihood ratio test, it is significant at *p* <.005

Table S3 DFGD Univariate Cox regression

Variable	HR	Cl_lower	Cl_upper	*p*
PaTRS	3.097	1.687	5.686	**0.000266**
Age	0.994	0.972	1.016	0.578
Sex				
Female	1			
Male	1.338	0.576	3.108	0.499
Hepatitis				
Hepatitis B	1			
Hepatitis C	1.465	0.202	10.599	0.705
Unknown	0.638	0.088	4.607	0.656
Antiviral_treatment				
No	1			
Yes	0.815	0.478	1.391	0.453
Smoke	0.78	0.15	4.055	0.768
No	1.435	0.363	5.672	0.607
Yes				
Alcohol	1			
No	0.844	0.475	1.499	0.563
Yes	1.481	0.896	2.448	0.125
Diabetes				
No	1			
Yes	0.705	0.256	1.942	0.499

Abbreviations: HR, hazard ratio. Cl_lower, Lower Confidience Limit . Cl_upper, Upper Confidience Limit. PaTRS, Patient level tumor risk scores.

The *p* was calculated based on likelihood ratio test, it is significant at *p* <.005

Table S4 DFGD Multivariate Cox regression

Variable	HR	Cl_lower	Cl_upper	*p*
PaTRS	3.284	1.735	6.216	**0.00026**
Age	0.993	0.969	1.018	0.577
Sex				
Female	1			
Male	1.175	0.477	2.898	0.726
Hepatitis				
Hepatitis B	1			
Hepatitis C	0.805	0.105	6.167	0.835
Unknown	0.362	0.048	2.746	0.326
Antiviral_treatment				
No	1			
Yes	0.745	0.433	1.282	0.288
Smoke				
No	1			
Yes	1.055	0.578	1.924	0.863
Alcohol				
No	1			
Yes	1.055	0.578	1.924	0.863
Diabetes				
No	1			
Yes	0.592	0.211	1.661	0.319

Abbreviations: HR, hazard ratio. Cl_lower, Lower Confidience Limit . Cl_upper, Upper Confidience Limit. PaTRS, Patient level tumor risk scores.

The p was calculated based on likelihood ratio test, it is significant at p <.005
